# The effect of *Lycium barbarum* on spinal cord injury, particularly its relationship with M1 and M2 macrophage in rats

**DOI:** 10.1186/1472-6882-13-67

**Published:** 2013-03-22

**Authors:** Yu-Kai Zhang, Jian Wang, Ling Liu, Raymond Chuen-Chung Chang, Kwok-Fai So, Gong Ju

**Affiliations:** 1 Institute of Neurosciences, Fourth Military Medical University, Chang Le Xi Road, Xi’an, Shan Xi, 710032, China; 2 Laboratory of Neurodegenerative Diseases, Department of Anatomy, LKS Faculty of Medicine, The University of Hong Kong, Pokfulam, Hong Kong SAR, China; 3 Research Centre of Heart, Brain, Hormone and Healthy Aging, LKS Faculty of Medicine, The University of Hong Kong, Pokfulam, Hong Kong SAR, China; 4 State Key Laboratory of Brain and Cognitive Sciences, The University of Hong Kong, Pokfulam, Hong Kong SAR, China; 5 Faculty of Medicine, The University of Hong Kong, 1/F Laboratory Block, 21 Sassoon Road, Pokfulam, Hong Kong SAR, China

**Keywords:** *L. barbarum*, Spinal cord injury, Macrophage, Rat

## Abstract

**Background:**

Our past researches suggested that *L. barbarum* exhibits direct neuroprotective and immune regulatory effects on the central nervous system, which are highly related to the events involved in the spinal cord injury, but not yet been investigated. Immune responses play an important role in the development of the pathology after secondary injury, particularly the M1 and M2 types of macrophage, on which special emphasis was laid in this study.

**Methods:**

In our previous studies *L. barbarum* was administrated orally from 7 days before the injury to ensure a stabilized concentration in the blood. For clinical application, *L. barbarum* can only be administered after the injury. Therefore, both pre-injury and post-injury administration protocols were compared. *In vivo* and *in vitro* studies were conducted and analyzed immunohistochemically, including Western blotting.

**Results:**

The lesion size in the pre-treated group was much larger than that in the post-treated group. To explain this difference, we first studied the effect of *L. barbarum* on astrocytes, which forms the glial scar encircling the lesion. *L. barbarum* did not significantly affect the astrocytes. Then we studied the effect of *L. barbarum* on microglia/macrophages, particularly the M1 and M2 polarization. After spinal cord injury, the deleterious M1 cells dominant the early period, whereas the beneficial M2 cells dominate later. We found that in the pre-treated group *L. barbarum* significantly enhanced the expression of M1 cells and suppressed that of M2 cells, while in the post-treated group LBP markedly promoted the activity of M2 cells. This explained the difference between the pre- and post-treated groups.

**Conclusions:**

*Lycium barbarum* has been wildly accepted to have beneficial effects in various central nervous system diseases. Our finding of deleterious effect of LBP administered at early period of spinal cord injury, indicates that its application should be avoided. The substantial beneficial effect of LBP when administered at later stage has an important impact for clinical application.

## Background

Spinal cord injury (SCI) has long been a focus of research, from both basic scientific and clinical therapeutic points of view [1,2]. We have previous studied early neurosurgery after spinal cord contusion on 30 ASIA-A (complete paralytic) patients [3]. All the patients had resumed certain degree of locomotion, the best results fell in a group of patients who were operated 4–14 days after SCI, in which 70% could walk with a pair of crutches or without any support. Immune response is one of the most important pathological events following SCI. Therefore, to further improve the clinical result, one of the crucial aspects is to investigate the changes and mechanism of immune responses after SCI. In SCI the immune response is a double edged sword [4-7], particularly the M1 and M2 types of macrophage [8]. In general, M1 is detrimental, whereas M2 is protective [5,7,8].

Traditional Chinese medicines have many benefits. *Lycium barbarum* (also named Fructus Lycii or Wolfberry), an upper class Chinese medicine in Chinese pharmacopoeia, is believed to be beneficial to the eye, kidney and liver and anti-aging. *Lycium barbarum* polysaccharide (LBP), the main content of *Lycium barbarum*, was found to serve most beneficial functions of wolfberry [9,10]. Our past researches suggested a direct neuroprotective effect on CNS of LBP, in models of Alzheimer's disease (Amyloid-β toxicity) [9-14], glutamate excitotoxicity [15], and Ocular hypertension [16-18], by down-regulation of c-Jun N-terminal signaling, RNA-dependent protein kinase phosphorylation, caspase-3 and caspase-2 activities, endoplasmic reticulum stress and up-regulation of Akt signaling or crystalline [11-13,15,17,18]. In Ocular hypertension model, we found a correlation between microglial activation and protection of RGC by LBP [16,17]. The immune regulation of LBP is also observed in mice and in human peripheral blood mononuclear cells [19-21]. It has been reported that LBP can be anti-oxidative [22-26].

The effects of LBP are highly related to SCI, but have not yet been studied. Its immune regulation and microglia/macrophage activation are particularly interesting, because of the importance of immune responses in SCI, especially the M1 and M2 types of macrophages. The aim of the present study was to investigate the effects of LBP in SCI and whether LBP could be a potential therapeutic medicine.

## Methods

### LBP administration

LBP was extracted as reported [16-18]. For animal experiments, the LBP was dissolved in warm double-distilled water, and fed through a nasogastric tube, 10 mg/kg, 2/day, throughout the experiment. For cell culture, 100 μg/ml of LBP was prepared and filtered through a 0.20 μm hole size microfilter (Dismic-25, Advantec, Stord, Norway).

### Animal grouping

Sixty male Sprague–Dawley (SD) rats (220 ~ 250 g), provided by the Animal Center of the Fourth Military Medical University, were maintained with temperature and light (12-h light/dark cycle) controlled. Food and water were provided ad libitum. Two plans of drug administration were designed. (1) In our previous studies, to ensure that the concentration of LBP in the blood had been stabilized at the time the insults happened, LBP was orally administered for 7 consecutive days in advance till the animals were sactificed. This was followed in the present study (LBP-pre group). (2) From clinical application of view, LBP can only be given after the injury. The administration of LBP started on the 7th day until the animals were sacrificed (LBP-aft group).

Animals were randomly divided into 6 groups. 1. LBP-pre group, 20 LBP treated and 20 vehicle control, with similar sub-grouping at 7 d and 14 d (n = 10, 6 for immunohistochemistry, 4 for western blotting). 2. LBP-aft group (14 d), 10 LBP treated and 10 vehicle control. In each of them, 6 rats for immunohistochemistry and 4 for western blotting.

The animal experiments were approved by the Animal Care Committee of the Fourth Military Medical University.

### In vitro study

Two types of cells were used: rat brain primary astrocyte of and murine N9 microglial cell line [27,28].

The primary astrocytes were isolated and purified from whole brain of newborn SD rat. Briefly, the brain was cut into small pieces and trypsinized to separate the cells, which were then cultured in 75 cm^2^ tissue culture flasks with DMEM medium containing 10% FBS, 100 μmol/L non-essential amino acids, 5 μg/ml insulin, 100 U/mL penicillin and 100 μg/mL streptomycin. When the cultured cells got confluent (7–10 d), the flask was shaken overnight (200 rpm at 37°C) to detach the microglia and oligodendrocytes. The remained cells were cultured 3 passages to purify the astrocytes. The purity of the primary astrocytes was determined by immunocytochemistry. Over 95% of the cells were GFAP immunoreactive. The primary astrocytes were seeded in identical density onto 10 mm circular slides in 24-well plate for immunocytochemistry, or directly into 6-well plate for western blotting, TNFα (20 ng/ml, Sigma, St. Louis, MO, USA), IFNγ (20 ng/ml, Sigma, St. Louis, MO, USA), LPS (200 ng/ml, Sigma, St. Louis, MO, USA) had been reported to induce astrocyte activation in vitro [29-32]. After incubating for 2 days with LBP, TNFα + IFNγ + LPS or TNFα + IFNγ + LPS + LBP, the level of GFAP expression was assessed. The incubation medium served as the negative control.

The murine N9 microglial cells, originally developed by P. Ricciardi-Castagnoli [33] were maintained in DMEM with 5% FCS. In experiment, cells were seeded in 6-well plate in an identical density. LPS (200 ng/ml) + IFNγ (20 ng/ml) and IL-4 (20 ng/ml, Sigma, St. Louis, MO, USA) were reported to induce M1 and M2 polarization of macrophages, respectively [5-7,34].

### Surgery

Tazlaff’s spinal cord lateral compression model [35] was followed. Briefly, a thin metal plate was attached onto the inner side of one blade of the forceps to leave 0.5 mm between the tips of the forceps when fully closed. The rats were anesthetized with 1% sodium pentobarbital (50 mg/kg) i.p. A 3~4 cm dorsal midline incision, centering at T8 vertebral spine, was made, followed by bilateral laminectomy of T8 vertebra to expose the spinal cord. The blades of the forceps were lowered along the lateral sides of the spinal cord till the vertebral canal was reached. The spinal cord was compressed in 8–10 seconds and remained in place for 20 seconds before the blades were let loose in 8–10 seconds and the forceps withdrawn. The skin incision was then closed. Manual evacuation of urinary bladder was performed twice daily until normal micturition resumed.

### Tissue preparation

The rats were sacrificed by an overdose of sodium pentobarbital (100 mg/kg) and perfused intra-cardially with 100 ml of warm normal saline followed by 400 ml 4% cold paraformaldehyde in phosphate buffer (pH 7.4). Following perfusion, a 2 cm spinal cord segment, with the injured site at the middle, was removed and put into 25% sucrose in phosphate buffer at 4°C until it sank. Serial 14 μm frozen sagittal sections were cut on a cryostat, and mounted on slides in 5 sets.

### Immunofluorescence staining

The immunostaining was conducted at room temperature. The tissue sections or cell culture slides were rinsed with 0.01 M PBS and then blocked with 1% BSA (Sigma) in PBS containing 0.5% Triton X-100 for 30 min. The sections were then incubated with primary antibody over night. After rinsed with PBS, the sections were incubated with the fluorescent second antibody for 2 h. Antibodies against markers of astrocyte (anti-GFAP, rabbit polyclonal, Dako Cytomaton, Denmark A/S, 1:5000, or), rat lysosomal membrane marker (anti-ED1, mouse monoclonal, Serotec, Raleigh, NC, USA; 1:400), iNOS (rabbit polyclonal, Cambridge, MA, USA, 1:50) and Arginase1 (goat polyclonal, Santa Cruz Biotechnology, Santa Cruz, California, USA,1:200) were used. The corresponding fluorescent secondary antibodies were purchased from Molecular Probes, Oregon, USA. Omission of the primary antibody served as the negative control. The sections were observed under Olympus BX-51 microscopy or FV1000 laser scanning confocal microscopy.

### Tissue section selection

The most important pathological changes after SCI occur in the main mass of the gray matter. In the central region of the spinal cord, there is only a narrow gray commissure, but there are many branches of blood vessels in the ventral fissure. The bleeding following crushing can be rather profuse and the extent of the blood squeezing along the dorsal funiclus is unpredictable, adding in a factor of individual variation of the data, thereby interferes the statistic analysis. In our pilot experiment, we succeeded in solving the problem by omitting the central sagittal sections and selected sections 200 to 600 μm from the central canal, where the main body of the gray matter was located. We followed this protocol in the present study.

### Microphotography for cultured cell

The primary astrocytes were cultured on 10 mm circular slide slides. By rotating 60°, 6 fields of each slide centering at the point 0.25 cm to the slide edge were micro-photographed under a 40 × objective.

### Measurement of lesion area

The lesion areas in the 7 d and 14 d groups were outlined along the inner edge of the GFAP strongly positive astrocytes. The areas were measured in photoshop CS3.

### Fluorescence intensity quantification

All the microphotos of an experiment group were taken with identical setting and the fluorescence intensity was measured.

For tissue sections, the integrated optical density (IOD) and area of the chosen region (AREA) were measured by Image-Pro Plus 6.0 and immunostaining intensity was expressed as IOD/AREA.

For primary astrocyte, the IOD and area of the whole picture (AREA) were measured, and the immunostaining intensity was calculated as above.

### Cell counting

In sections triple-stained with GFAP, ED1 and iNOS or Arg1, cell counting was performed in the area within 1 mm rostral or caudal to the lesion edge. On each side (rostral or caudal) in each section, ED1^+^iNOS^+^ or ED1^+^iNOS^+^ cells were counted in areas of 250 × 250 μm. Due to the irregularity of the distal edge, at least three squares could be chosen at random. The cell counts were normalized to mm^2^ cord section.

### Western blotting

For protein samples from the spinal cord, a 0.5 mm segment of the spinal cord, centering at the injury site, was dissected out from the sacrificed animal. The cord was placed in cold lysis buffer and homogenized on ice using a tissue homogenizer (Tissue Tearor 985370, Biospec Products, Bartlesville OK, USA). The samples were centrifuged (12000 RMP) 4°C for 5 min. Afterwards, the supernatants were flashly frozen and stored at −80°C until used.

For cultured microglia or astrocyte, 200 μL cold lysis buffer was added into the well of 6-well plate for harvesting. The samples were homogenized with a 200 ml transferpettor, and then treated as described above.

The samples were boiled for 5 min in strong denaturing conditions, loaded on SDS-polyacrylamide gels, and transblotted onto polyvinylidene difluoride membranes (Millipore Corporation, Bedford, MA). The blots were blocked with 1% defatted milk for 1 h at room temperature and incubated overnight at 4°C with the following antibodies: anti-GFAP (rabbit polyclonal, Anbo, San Francisco, C.A., USA, 1:800), anti-ED1 (mouse monoclonal, Serotec, Raleigh, NC, USA, 1:400), anti-iNOS (rabbit polyclonal, Cambridge, MA, USA, 1:500), anti-Arginase1 (goat polyclonal, Santa Cruz Biotechnology, Santa Cruz, California, USA,1:1000). After incubation with an HRP-conjugated secondary antibody, the samples were subjected to enhanced chemiluminescence and densitometric analysis. Band densitometric analysis was performed in Image-Pro Plus 6.0.

### Data analysis

The mean value of selected sections in each rat or selected images on each slide was calculated for statistic analysis. Each in vitro experiment was repeated 3 independent times at least. For western blotting results, change of abundancy of protein was calculated by measuring IOD of a protein divided by IOD of the corresponding β-actin. All data was analyzed using SPSS 12.0. Unpaired student's *t*-test was used to compare each treated samples with the corresponding controls, and Correlation regression for correlation analysis. Statistic results were shown as mean ± standard error. P values less than 0.05 (p < 0.05) was taken as significant.

## Results

### Effect of LBP on lesion area

**Figure 1:**
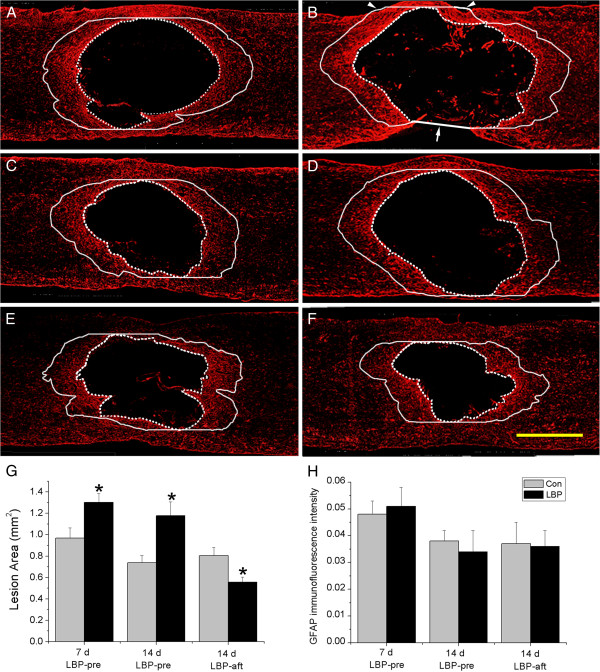
**Lesion size and immunofluorescence intensity A-F.** GFAP immunostaining. **G**. Lesion size. **H**. Immunofluorescence intensity. The lesion area is delineated along the inner boundary of the GFAP immunostained astrocytic scar (dash line). The immunofluorescence was evaluated in the areas 0.5 mm rostral and caudal to the lesion (solid line). In **B** the ventral part of the spinal cord was broken. A straight line between the broken ends was drawn (arrow). Also, at the upper right and left parts (arrow heads) there were two small parts where the solid line was drawn outside of the section. The areas of these two small parts were measured. They covered less than 1% of the entire interested area, and were neglected in the evaluation. **A**-**D**. LBP-pre group: **A**, **C**. 7 d and 14 d vehicles; **B**, **D**. 7 d and14 d corresponding LBP treated cases. At both 7 d and 14 d the lesion sizes were significantly larger than that of the control (**G**), but there was no significant difference in the immunofluorescence intensity of GFAP (**H**). **E**, **F**. LBP-aft 14 d group. Unlike the LBP-pre group, the lesion area was smaller in the drug group. Similar to the LBP-pre group there was no significant difference in the immunofluorescence intensity between the control and LBP treated groups. *p < 0.05, compared to the corresponding vehicle control. Bar = 1 mm.

In the LBP-pre group the lesion size at 7 d and 14 d was 1.30 and 1.17 mm^2^, respectively. In the vehicle group, they were 0.97 and 0.75 mm^2^, respectively. The differences between LBP and vehicle were statistically significant at 7 d (p = 0.028) and 14 d (p = 0.014) (Figure 1A-D, G). In the LBP-aft group the lesion size was 0.56 mm^2^ at 14 d, and in the vehicle group 0.80 mm^2^. The difference was statistically significant (p = 0.031) (Figure 1E-G). The results demonstrated that the lesion sizes in the LBP-pre group were larger than that of the LBP-aft group (p = 0.0026). In short, LBP could reduce the size of the lesion site; administer of LBP before the spinal cord injury markedly increased the lesion. It occurred to us that the difference between the lesion sizes of LBP-pre and LBP-aft could be due to the effect of LBP on astrocytes. Astrocytes form the major part of the glial scar, which, as a protective measure, limits the lesion site from expansion. If LBP could inhibit the astrocytes, the glial scar would be weakened.

### Effect of LBP on astrocyte

#### In vivo study

At 7 d and 14 d after SCI, the GFAP immunofluorescence intensity of the astrocytes in the area 0.5 mm rostral and caudal to the lesion edge, where most of the reactive astrocytes in the gray matter were located, was evaluated. There was no significant difference between the LBP-pre, LBP-aft groups, and their respective vehicles, indicating that LBP does not have influence on astrocytes in SCI (Figure 1A-F and, H).

#### In vitro study

Given the complexity of *in vivo* environment that may counteract the deleterious effect on astrocytes, e.g. M2 macrophage, we conducted *in vitro* study to verify it.

**Figure 2:**
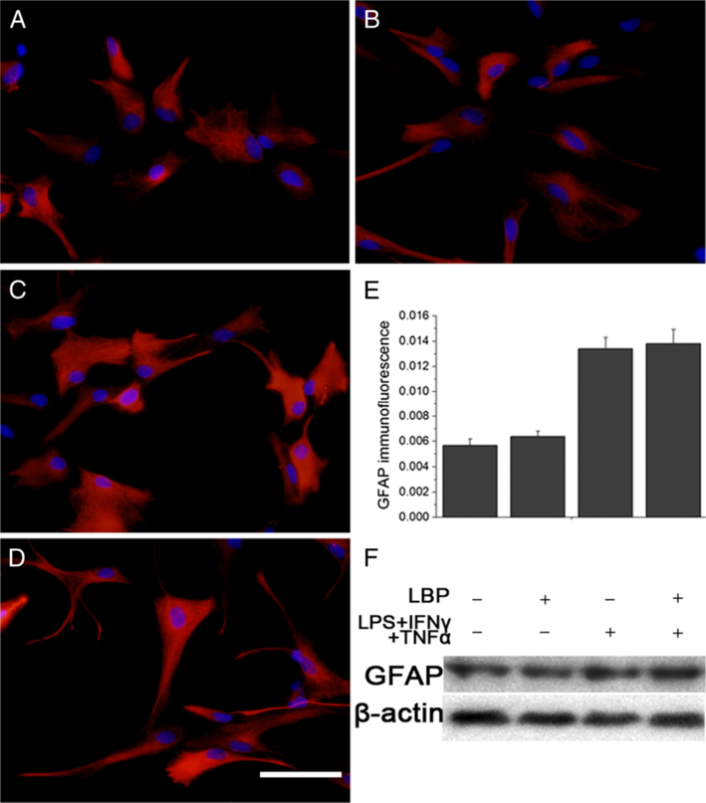
**Effect of LBP on GFAP expression in primary astrocyte.** Astrocyte stimulated with TNFα + IFNγ + LPS displayed hypertrophic appearance (**C**) and higher GFAP expression (**E**, **F**), compared to the vehicle group (**A**). Cells treated with LBP (**B**) or LBP plus TNFα + IFNγ + LPS (**D**) showed no difference in staining intensity (**E**) and GFAP level (**F**), versus the corresponding controls, respectively (**A**, **C**). Bar = 50 μm.

The TNFα + IFNγ + LPS induced strong GFAP immunoreactivity in the cultured primary astrocytes, whereas the vehicle treated astrocytes showed much weaker immunoreactivity. The LBP plus TNFα + IFNγ + LPS and LBP induced no significant difference in GFAP level, compared to TNFα + IFNγ + LPS and vehicle group, respectively (Figure 2). The *in vitro* study lent support to the *in vivo* study.

Both the *in vivo* and *in vitro* studies demonstrated that LBP has no effect on astrocytes. We then thought, if the difference could be due to their effect on microglia/macrophage.

### Effect of LBP on microglia/macrophage

#### Changes of ED1

##### Western blotting

**Figure 3:**
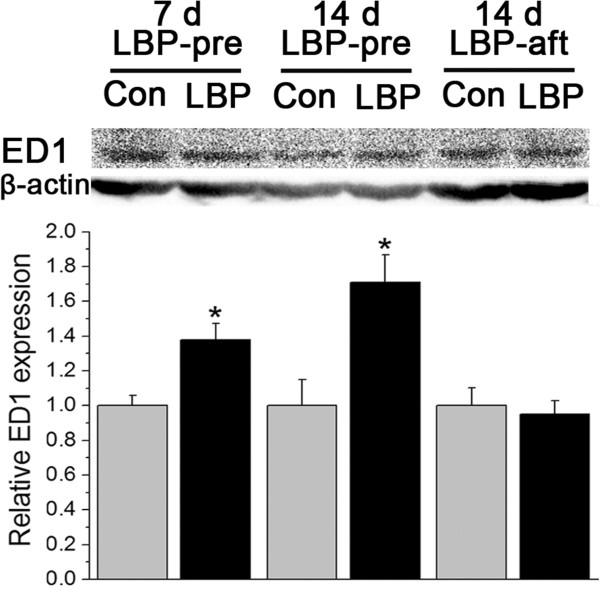
**Effects LBP on ED1 expression ED1 levels were normalized to the corresponding vehicle controls at the same time point in the same group.** LBP-pre groups exhibited an increased ED1 expression, relative to the controls at corresponding time. No measurable difference was found between LBP-aft group and their control. *p < 0.05, compared to the corresponding vehicle control.

In the LBP-pre 7 d and 14 d groups the expressions of ED1 were higher than their respective controls (p = 0.013 at 7 d, 0.017 at 14 d). In the LBP-aft group there was no difference in the expressions of ED1 between the drug and control (p = 0.71) (Figure 3).

##### Immunohistochemistry

**Figure 4:**
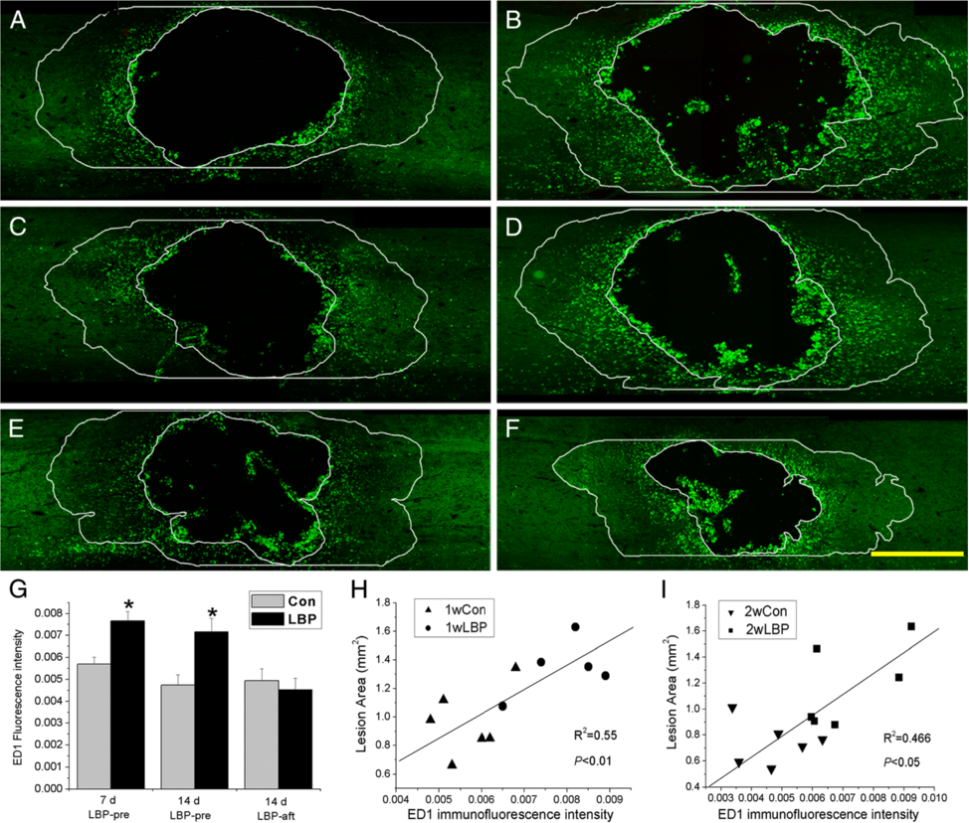
**Correlation between change of ED1 expression and lesion area.** ED1 fluorescence intensity in area outside lesion and within 1 mm to the edge (two bands rostral and caudal to lesion, outlined by lines on each section) was measured, with the positive labeling within lesion out of our calculation. Animals given LBP-pre exhibited increased ED1 immunofluorescence intensity at either 7 d (**B**) or 14 d (**D**), compared to their corresponding vehicle groups, respectively (**A**, 7 d, **C**, 14 d). And in these animals, the increased ED1 staining intensity correlated with lesion change induced by LBP, at either 7 d (**H**) or 14 d (**I**). In rats treated with LBP-aft, no significant difference was found between LBP group (**F**) and control (**E**), at 14 d after injury. *p < 0.05, compared to the corresponding vehicle control; Bar = 1 mm. (**G**) ED1 fluorescence intensity of LBP-pre and LBP-aft groups.

The microglia and macrophages were labeled with antibody against ED1. The ED1 positive cells were predominantly distributed around the injury site. Their immunoreactivity declined farther away from the lesion (Figure 4A-F), in the area 1 mm rostral and caudal to the lesion edge, the ED1 fluorescence intensity in the LBP-pre group was increased by 34% at 7 d (Figure 4A, B) and 51% 14 d (Figure 4C, D) compared to their respective vehicle group (p = 0.0037 at 7 d, 0.01 at 14 d) (Figure 4G). The correlation coefficients between the fluorescence intensity and the lesion size were 0.74 at 7 d (p = 0.004) (Figure 4H) and 0.68 at 14 d (p = 0.014) (Figure 4I). There was no significant change in ED1 immunostaining intensity between the LBP-aft group and the vehicle (p = 0.35) (Figure 4E, F, G).

Thus, both the Western blotting and immunohistochemical studies suggested that even the lesion size was highly correlated with the increase in ED1 immunoreacivity, it was not the result of LBP administration. We then turn to investigate whether it was their effect on M1 and M2 cells that makes the difference.

### Effect of LBP on M1 and M2

#### In vivo study

##### Western blotting

**Figure 5:**
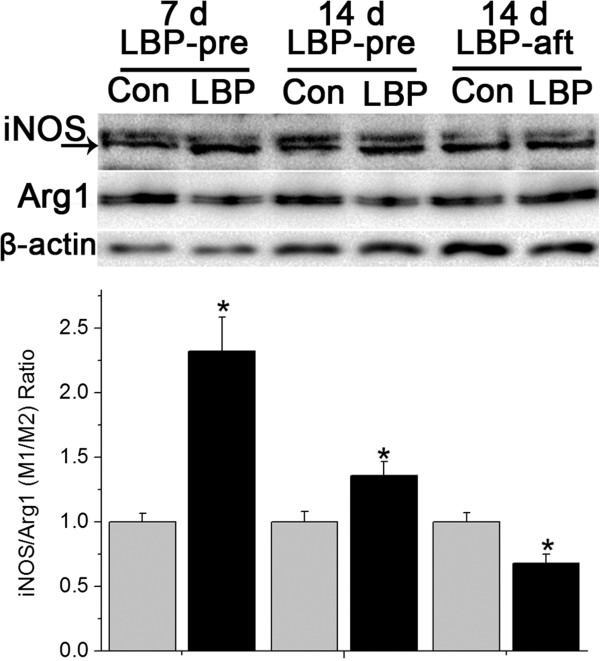
**Effects LBP on microglia/macrophage activation (polarization) in SCI.** Relative numbers of iNOS/Arg1 ratio to the corresponding vehicle controls at the same time point in the same group were shown. In rats treated with LBP-pre, iNOS was up-regulated and Arg1 down-regulated, with consequent higher ratio of iNOS/Arg1, compared to vehicle groups, at both 7 d and 14 d, respectively. Whereas, LBP-aft administration induced both increase in iNOS and Arg1 level, but finally got a reduced iNOS/Arg1 ratio, versus its vehicle control at 14 d. *p < 0.05, compared to the corresponding vehicle control.

The effect of LBP on M1 and M2 was evaluated by the changes of iNOS and Arginase1 (Arg1) expressions. The expressions of iNOS in the LBP-pre 7 d and 14 d groups were higher than their respective controls. The Arginase1 levels in the corresponding groups were all decreased. The ratio between the iNOS and Arg1 rose by 1.35 fold at 7 d and 36% at 14 d. In the LBP-aft group at 14 d, both the iNOS and Arg1expressions were up-regulated, however, the increment of Arg1 was greater than that of iNOS, leading to a reduction of their ratio by 32% (Figure 5).

##### Immunohistochemistry

**Figure 6:**
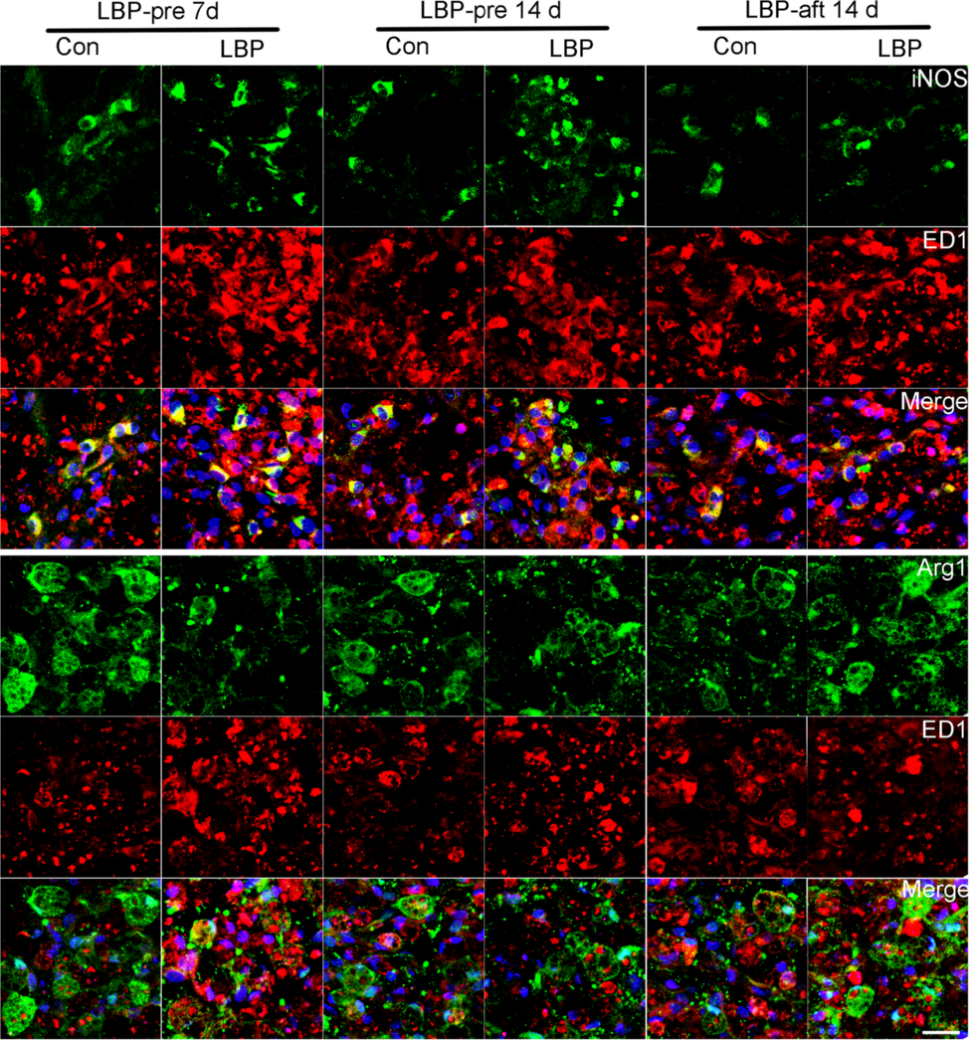
**Quantitative evaluation of the M1 and M2 macrophage/microglia.** By immunofluorescence staining, most iNOS or Arg1 were expressed in ED1 positive cells. In LBP-pre groups at 7 d and 14 d, there were elevated ED1 expression, increased ED1^+^iNOS^+^ cells and reduced ED1^+^Arg1^+^ (M2) cell, in comparison to the controls. LBP-pre treated animals showed an increased number of M2 cells. Bar = 20 μm.

**Figure 7:**
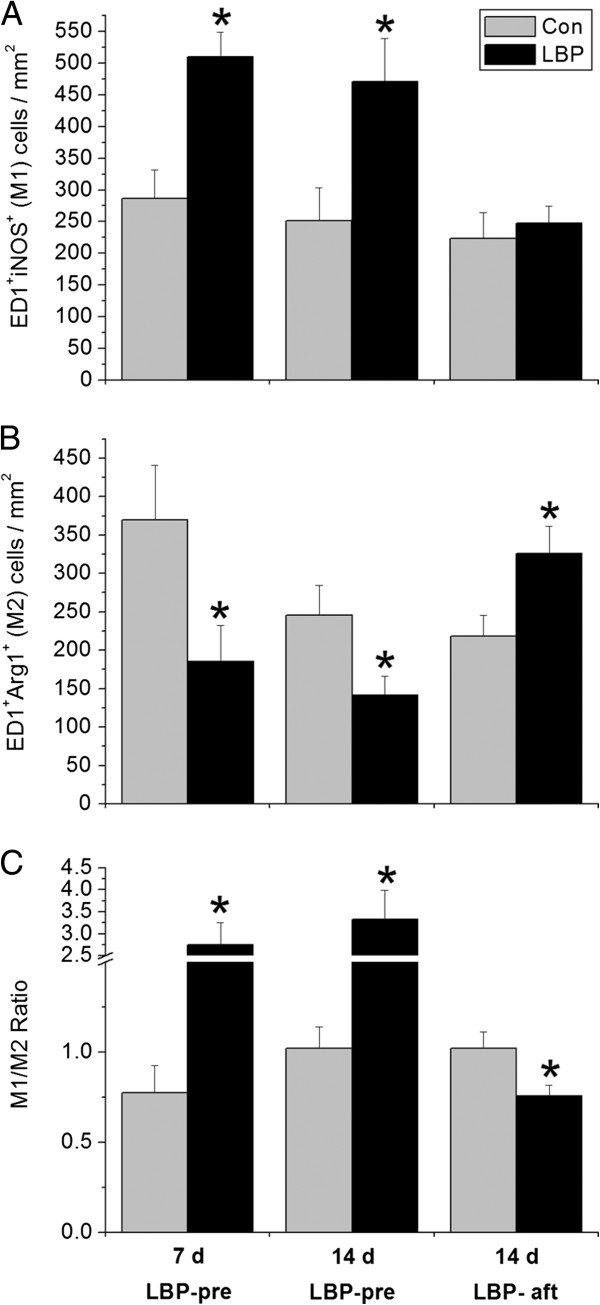
**Statistic result of the number of M1 and M2 macrophage/microglia and M1/M2 ratio.** LBP-pre groups showed increased M1 (**A**), reduced M2 cells (**B**) and a remarkable higher M1/M2 ratio (**C**), relative to their corresponding controls. LBP-aft animals exhibited no difference in M1 number (**A**) however increased M2 cells (**B**), resulting a significantly lower M1/M2 ratio (**C**), versus the controls. *p < 0.05, compared to the corresponding vehicle controls.

Since it has been reported that iNOS can be expressed in neurons and neutrophils, immunohistochemistry was used to confirm if the iNOS/Arg1 changes represent the M1/M2 ratio. ED1^+^ cells expressing iNOS or Arg1 in the area 1 mm rostral and caudal to the lesion edge were counted. The LBP-pre animals at 7 d and 14 d showed increased ED1^+^iNOS^+^ (M1) and reduced ED1^+^Arg1^+^ (M2) cells, exhibiting significantly higher M1/M2 ratio, relative to the corresponding controls. In LBP-aft study, no measurable changes was found in M1 cell number between the two groups, however, treated animals displayed an increased number of M2 cells, with a resultant lower M1/M2 ratio (Figures 6, 7).

##### In vitro study

The N9 microglial cell line was used. Various M1 activators, LPS + IFNγ, and M2 activator, IL-4, were applied. iNOS and Arg1 were used as M1 and M2 markers, respectively, the results were evaluated with Western blotting.

**Figure 8:**
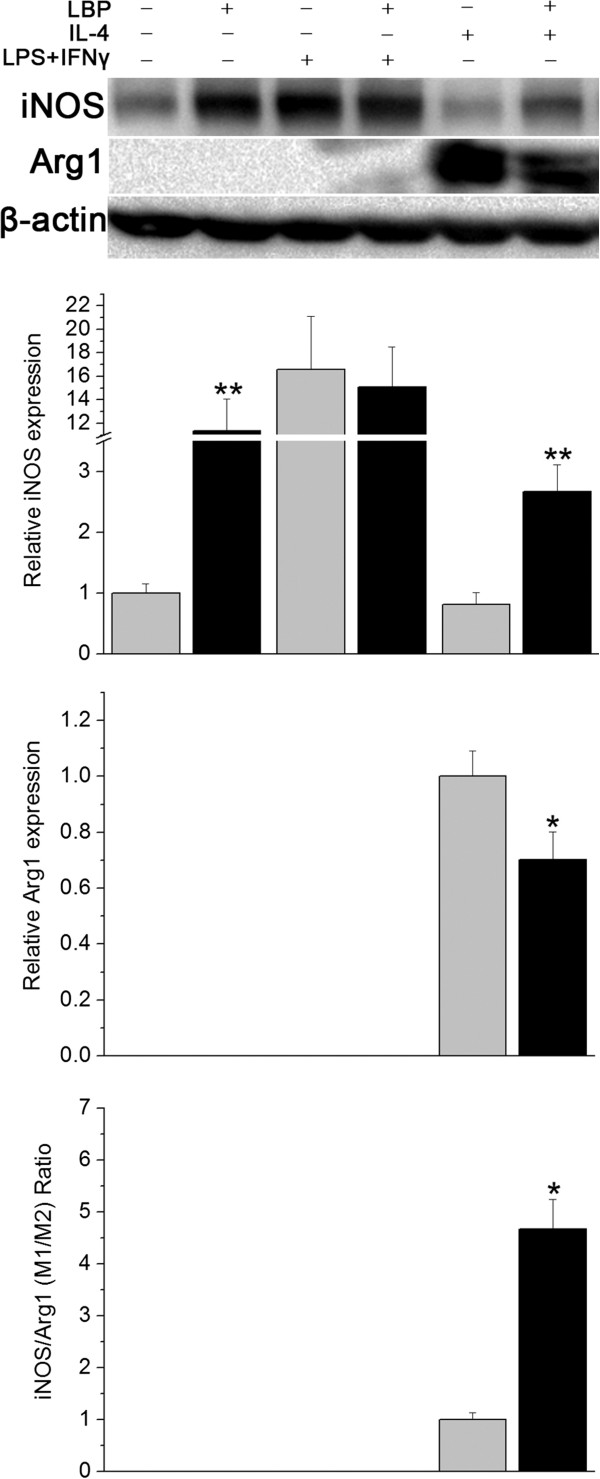
**Effects of LBP on murine N9 microglial cell N9 cells were treated with different medicines.** The iNOS level was shown as relative number to that of the vehicle group, and the Arg1 level and iNOS/Arg1 ratio were normalized to that of IL-4 group. Vehicle treated N9 cells displayed mild iNOS and little Arg1. LPS + IFNγ and IL-4 induced markedly enhanced M1 and obvious M2, respectively. LBP alone and allied with IL-4 led to increased iNOS and suppressed Arg1 expression, compared to vehicle and IL-4, respectively. *p < 0.05, **p < 0.01, compared to the corresponding controls treated with same medicine short of LBP.

The first part of the experiment was to study the effect of LBP, including LBP and the vehicle, LBP plus LPS + IFNγ and LPS + IFNγ, LBP plus IL-4 and IL-4, six groups. It had been demonstrated that LBP up-regulated iNOS, but no Arg1expression could be detected. The vehicle showed only mild level of iNOS. The LBP plus LPS + IFNγ and LPS + IFNγ groups displayed similar results as the LBP group. IL-4 caused decrease in iNOS and increase in Arg1 and LBP plus IL-4 induced a reversal of IL-4 effects (Figure 8).

To investigate the difference between the LBP-pre and LBP-aft groups, two experiments were designed. For LBP-pre, the N9 cells were incubated with LBP on the first one day and LBP plus LPS + IFNγ + IL-4 on the next day. Omission of LBP served as the control. For LBP-aft, the N9 cells were treated with LPS + IFNγ + IL-4 on the first day and on the next day the cells were treated with LBP plus LPS + IFNγ + IL-4 or the control LPS + IFNγ + IL-4.

**Figure 9:**
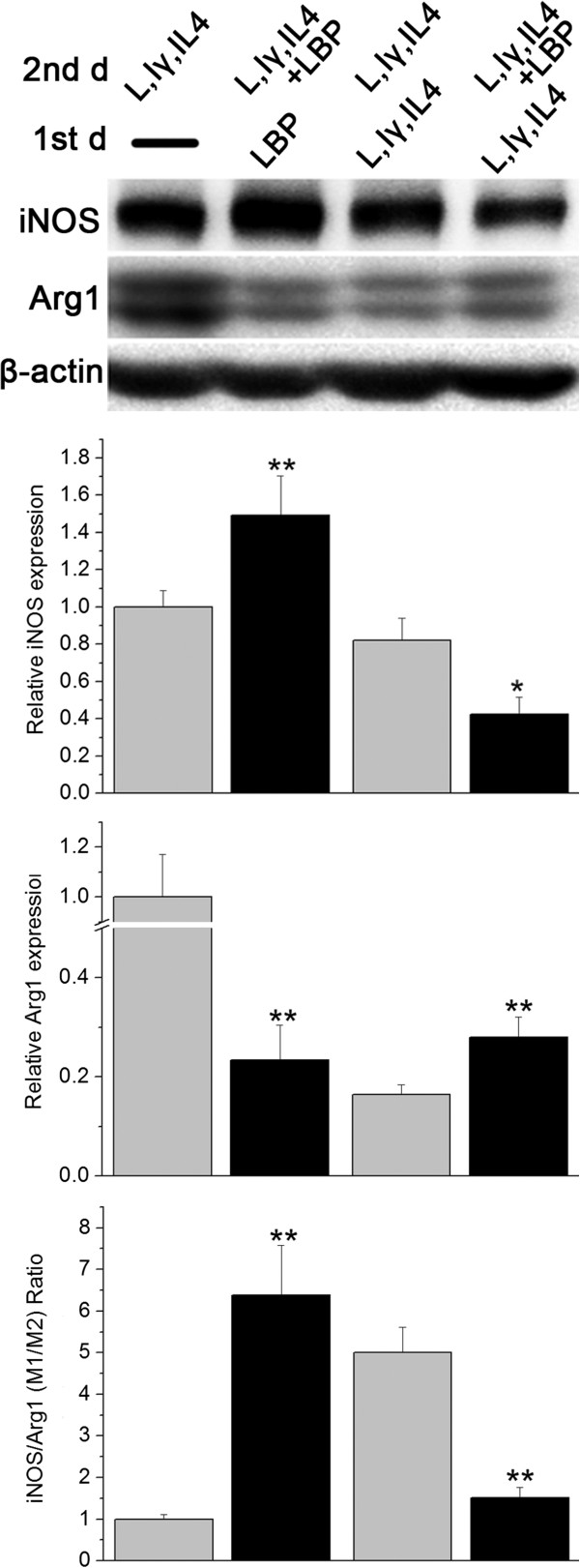
**The in vivo LBP-pre and LBP-aft study was mimicked in N9 cells.** N9 cells were treated with different medicines for 2 days. Relative numbers to the first group were shown. N9 cells were incubated with LBP for first one day and LBP plus LPS + IFNγ + IL-4 for next day, and presented a higher M1 and suppressed M2 polarization compared to its LBP-free control. If the cells were treated with LPS + IFNγ + IL-4 for first day, and the LBP plus LPS + IFNγ + IL-4 for the second, LBP group displayed reduction in iNOS and elevation in Arg1 expression in comparison to its control that stimulated with LPS + IFNγ + IL-4 for corresponding 2 days. *p < 0.05, **p < 0.01, compared to the corresponding controls treated with same medicine but lack of LBP. L,Iγ,IL4, abbreviation for LPS + IFNγ + IL-4.

In the LBP-pre study, the iNOS expression was elevated and Arg1 reduced. The M1/M2 ratio was higher than its LBP free control. In LBP-aft, the iNOS expression was reduced and Arg1 elevated. The M1/M2 ratio was lower than the LBP free control (Figure 9).

## Discussion

The lesion size in the LBP-pre group was larger than that in the LBP-aft group came as a surprise to us and we had to face the challenge of finding out the explanation.

Since the astrocytic scar is important in limiting the lesion size [36-38], we first examined the effect of LBP on astrocytes. The result of our *in vitro* study demonstrated that LBP had no effect on astrocytes. We then turn to study the effects of the LBP-pre and LBP-aft groups on microglia/macrophage. Our ED1 immunohistochemical study showed that, though the immunointensity was highly correlated with the lesion size, the enhanced immunointensity was not the result of LBP administration. There are reports demonstrating that after SCI the macrophages response actively and play an important role in the development of secondary injury [5-7]. Furthermore, M1 produces high level of oxidized metabolic substances, such as NO, superoxides, pro-inflammatory factors, IL-12, IL-1β, IL-15, IL-18, TNF-α, CCL15, and CCL20. Furthermore, its expression of chondroitin sulphate proteoglycan is 17 times higher than that on M2 cells. On the contrary, M2 secretes anti-inflammatory factor IL-10 and TGF-β. In addition, they can secrete various neurotrophines, like NGF, BDNF, NT-3 and neurotrophic factors, including CNTF, IGF, EGF, PDGF, GDNF [39,40]. M1 can inhibit neurite outgrowth of dorsal root ganglion, while M2 has the opposite effects. In vitro evidence shows that M1 macrophage can directly induce neuronal death [41-43]. We then, therefore, focused on the changes in M1 and M2 macrophages. Our data showed that M1 dominated right after the insult and gradually sifted to M2 dominance. LBP treatment before the injury enhances M1 and suppresses M2, whereas LBP administered 7 days after the injury increased the number of M2 cells.

*Lycium barbarum* has been wildly accepted to have beneficial effects in various central nervous system illness. Our finding of deleterious effect of LBP administered at early period of spinal cord injury, so that its application should be avoided, and the substantial beneficial effect of LBP when administered at later stage has an important impact for clinical application.

## Conclusions

Our previous studies have demonstrated that LBP has many beneficial effects in the central nervous system, including retina. But its effect had never been studied in spinal cord injury. The present study found that major effect of LBP in spinal cord injury is via its action on M1 and M2 types of macrophages. It is well known that M1 macrophages is deleterious, which dominate the first period of spinal cord injury and gradually shift to M2 dominance, which is benificial. Administration of LBP at the beginning of the injury enhances M1 and suppresses M2, whereas administration of LBP from 7th day on after injury has significant beneficial effect in reducing the secondary injury. This finding is an important issue that is valuable for its translation to clinical application.

## Competing interests

The authors have no competing interests to declare.

## Authors’ contributions

Conceived and designed the experiments: KFS, GJ. Performed the experiments: YKZ, LL. Analyzed the data: YKZ, JW, KFS, GJ. Contributed reagent: RCCC. Wrote the paper: YKZ, JW, GJ. All authors read and approved the final manuscript.

## Pre-publication history

The pre-publication history for this paper can be accessed here:

http://www.biomedcentral.com/1472-6882/13/67/prepub
